# Utilizing an Animal Model to Identify Brain Neurodegeneration-Related Biomarkers in Aging

**DOI:** 10.3390/ijms22063278

**Published:** 2021-03-23

**Authors:** Ming-Hui Yang, Yi-Ming Arthur Chen, Shan-Chen Tu, Pei-Ling Chi, Kuo-Pin Chuang, Chin-Chuan Chang, Chiang-Hsuan Lee, Yi-Ling Chen, Che-Hsin Lee, Cheng-Hui Yuan, Yu-Chang Tyan

**Affiliations:** 1 Department of Medical Education and Research, Kaohsiung Veterans General Hospital, Kaohsiung 813, Taiwan; w3e3@hotmail.com (M.-H.Y.); plchi@vghks.gov.tw (P.-L.C.); 2 Graduate Institute of Biomedical and Pharmaceutical Science, Fu Jen Catholic University, New Taipei City 242, Taiwan; arthur@kmu.edu.tw; 3 Department of Medical Imaging and Radiological Sciences, Kaohsiung Medical University, Kaohsiung 807, Taiwan; shan1102112@gmail.com; 4 International Degree Program in Animal Vaccine Technology, International College, National Pingtung University of Science and Technology, Pingtung 912, Taiwan; kpchuang@g4e.npust.edu.tw; 5 Research Center for Animal Biologics, National Pingtung University of Science and Technology, Pingtung 912, Taiwan; 6 Graduate Institute of Animal Vaccine Technology, College of Veterinary Medicine, National Pingtung University of Science and Technology, Pingtung 912, Taiwan; 7 School of Medicine, College of Medicine, Kaoshiung Medical University, Kaoshiung 807, Taiwan; chinuan@gmail.com; 8 Department of Nuclear Medicine, Kaohsiung Medical University Hospital, Kaohsiung 807, Taiwan; 1010.yiling@gmail.com; 9 Department of Electrical Engineering, I-Shou University, Kaohsiung 840, Taiwan; 10 School of Medicine, College of Medicine, Kaohsiung Medical University, Kaohsiung 807, Taiwan; 11 Neuroscience Research Center, Kaohsiung Medical University, Kaohsiung 807, Taiwan; 12 Department of Nuclear Medicine, Chi Mei Medical Center, Tainan 710, Taiwan; chlee4@ms45.hinet.net; 13 Department of Biological Sciences, National Sun Yat-sen University, Kaohsiung 804, Taiwan; chlee@mail.nsysu.edu.tw; 14 Mass Spectrometry Laboratory, Department of Chemistry, National University of Singapore, Singapore 119077, Singapore; chmyuch@nus.edu.sg; 15 Graduate Institute of Medicine, College of Medicine, Kaohsiung Medical University, Kaohsiung 807, Taiwan; 16 Institute of Medical Science and Technology, National Sun Yat-Sen University, Kaohsiung 804, Taiwan; 17 Department of Medical Research, Kaohsiung Medical University Hospital, Kaohsiung 807, Taiwan; 18 Center for Cancer Research, Kaohsiung Medical University, Kaohsiung 807, Taiwan; 19 Research Center for Environmental Medicine, Kaohsiung Medical University, Kaohsiung 807, Taiwan

**Keywords:** GNMT, aging, biomarker, neurons

## Abstract

Glycine N-methyltransferase (GNMT) regulates S-adenosylmethionine (SAMe), a methyl donor in methylation. Over-expressed SAMe may cause neurogenic capacity reduction and memory impairment. GNMT knockout mice (GNMT-KO) was applied as an experimental model to evaluate its effect on neurons. In this study, proteins from brain tissues were studied using proteomic approaches, Haemotoxylin and Eosin staining, immunohistochemistry, Western blotting, and ingenuity pathway analysis. The expression of Receptor-interacting protein 1(RIPK1) and Caspase 3 were up-regulated and activity-dependent neuroprotective protein (ADNP) was down-regulated in GNMT-KO mice regardless of the age. Besides, proteins related to neuropathology, such as excitatory amino acid transporter 2, calcium/calmodulin-dependent protein kinase type II subunit alpha, and Cu-Zn superoxide dismutase were found only in the group of aged wild-type mice; 4-aminobutyrate amino transferase, limbic system-associated membrane protein, sodium- and chloride-dependent GABA transporter 3 and ProSAAS were found only in the group of young GNMT-KO mice and are related to function of neurons; serum albumin and Rho GDP dissociation inhibitor 1 were found only in the group of aged GNMT-KO mice and are connected to neurodegenerative disorders. With proteomic analyses, a pathway involving Gonadotropin-releasing hormone (GnRH) signal was found to be associated with aging. The GnRH pathway could provide additional information on the mechanism of aging and non-aging related neurodegeneration, and these protein markers may be served in developing future therapeutic treatments to ameliorate aging and prevent diseases.

## 1. Introduction

Aging involves cells which cease dividing or an organism’s tissues/organs to decline physically and functionally. Thus, accelerated degenerative diseases are often age-related and time-dependent. Aging-associated medical conditions, to name a few, include atherosclerosis and cardiovascular disorders, diabetes, cataracts, osteoporosis, hypertension, cancer, and neurodegenerative diseases, such as Alzheimer’s disease [1]. Hence, a study of how aging progresses, what aging affects, and prevention becomes crucial. Nine general cellular and molecular characteristics of aging have been categorized as follows: genomic instability, telomere attrition, epigenetic alterations, loss of proteostasis, deregulated nutrient sensing, mitochondrial dysfunction, cellular senescence, stem cell exhaustion, and altered intercellular communication [2]. Having one or more dysfunction from the above categories may result in illness, but focusing on solving one specific type of problem may result a contradiction with other hallmarks, as the complicated biomolecular mechanisms that contributed to the aging process are not well-known. The intervened mechanisms or pathways should be carefully addressed when working on the aging problem.

Aging causes the level of circulating sarcosine to be reduced, which can be up-regulated by the enzyme of glycine N-methyltransferase (GNMT) [3]. Recently, an article showed that using GNMT to enhance S-adenosyl-methionine (SAM) catabolism, similar to inhibition of SAM synthesis, can extend the lifespan of Drosophila [4]. The enzyme GNMT catalyzes the process of methylation which is SAM-dependent. It was suggested that GNMT regulates the amounts of S-adenoslhomocystine (SAH) and SAM in which SAM is a major methyl donor for cellular methylation reactions, and thus GNMT is likely to have the capacity to regulate cellular methylaton [5,6]. In addition, GNMT is abundant in the liver and has been found in pancreas, kidney, and neuron cells, which indicates that GNMT may be involved in secretion [5,7,8]. In Obata’s study, similar to previous findings, GNMT happened to be a predominant regulator of systemic SAM expression level, which was increased during aging but can be maintained under pro-longevity regimens [4]. The systemic SAM levels in flies are increasing when aged, but up-regulated expression of GNMT can suppress this age-reliant SAM increase and thus extends longevity. The lifespan of Drosophila flies was extended due to the enhancement of SAM catabolism by GNMT. This indicates that the age-dependent SAM increase was weakened using lifespan-extending regimens. They also showed that GNMT was involved in the central role for such regimens and its over-expression increased longevity [4]. Thus, GNMT may play an important role in anti-aging where deficiency of GNMT may accelerate aging-related disorders.

Modulators for neurogenesis include pathways of signaling transduction, systems of immune and epigenetic regulation, and metabolic factors [9]. Neural stem cells (NSCs) can undergo proliferation and differentiation, which are regulated through several signaling pathways, such as Wnt or Notch. Any alterations of modulators during adult neurogenesis could lead to neurodegenerative disease developments. Biomarkers are physiological responsive signals that can be used as indicators of experiencing stress within an individual. Proteins, including missing proteins, changed in quality or quantity according to particular biological events, fall into this category since measurements of changes are essential to the biomarker discovery [10,11]. Missing proteins are proteins not evident at the protein level due to informatics, technical issues or biological expression patterns [12]. They have roles in various cellular activities in both growing and aging conditions. Thus, missing proteins in aged animal models represent potential biomarkers associated with aging, both physiological and pathophysiological. Additionally, aging-specific protein biomarkers could help us recognize aging pathways where proteins participate heavily. Besides, GNMT can influence genetic stability through DNA methylation, and the loss of GNMT impairs nucleotide biosynthesis [13]. Neurons’ GNMT expression in the brain has been detected from the cerebral cortex, hippocampus, substantia nigra and cerebellum [14]. Thus, GNMT may be crucial in normal physiological brain function, and GNMT-knockout mice (GNMT-KO) can be a useful model to study signaling pathway dysfunction [14]. The molecular process and biomarker of aging may be the key to prevent, delay, or alleviate age-associated diseases. In this study, we utilized proteomic techniques to characterize neurogenesis-related proteins involved in aging. Understanding the function of these signaling proteins may show the way for new treatments of human neurological disorders.

## 2. Materials and Methods

### 2.1. Mouse GNMT Isolation and Generation

Wide-type (Wt) C57BL/6 mice were purchased from the National Laboratory Animal Center (NLAC) in Taiwan. A C57BL/6-strain mouse placental genomic DNA library constructed in lambda phage FIX II (Stratagene, La Jolla, CA, USA) was used to isolate GNMT genomic clones. Human GNMT complementary DNA was used as a probe; hybridization procedures were performed according to standard protocols. A targeting vector was constructed and used to generate the GNMT knockout mouse model. All mice were kept in a 12-h light-dark-cycle room with water and standard mouse pellet chow. All the mice had been fasting for at least 8 h before sacrifice. Each experiment was composed of eight to ten sets of animals. All animal experiments were carried out in accordance with the National Institutes of Health (NIH) guidelines for the Care and Use of Laboratory Animals and approved by the Institutional Animal Care and Use Committee of Kaohsiung Medical University and performed in compliance (IACUC #108210). Three to four-month-old male and 24 to 30-month old male Wt C57BL/6 and GNMT^−/−^ mice were sacrificed (*n* = 8/group).

### 2.2. Protein Confirmation-Western Blotting

To validate the proteins related to aging and damage in the brain, Western blotting was applied to detect candidate proteins that may be related to aging. Protein extracts from whole brain lysates were prepared in lysis buffer and each protein sample (1 μg/μL, 10 μL) is electrophoresed through a precast gel (NuPAGE^®^Novex^®^ 4–12% Bis-Tris Gel, 1.5 mm, 10 wells, Invitrogen^TM^, Carlsbad, CA, USA.).Proteins were transferred from the gel to a polyvinyldifluoride (PVDF) membrane by means of the semidry technique using the Criterion Blotter (Bio-Rad, Hercules, CA, USA) at 100 V for 60 min, and blocked with 5% milk in PBS (adjusted to pH 7.4) containing 0.05% Tween-20. The membranes were then separately incubated overnight with primary antibodies (1 μg/μL). After washing, the membrane was incubated with HRP-conjugated secondary antibodies for one hour (1:10,000). Proteins were detected with an enhanced chemiluminscent (ECL) system, and quantitative analysis of Western blotting is carried out using the ImageQuant-TL7.0 software (GE Healthcare, Chicago IL, USA).

### 2.3. Tissue Preparation for Histology and Immunohistochemistry

Brain hemispheres, in pairs, were paraffin-embedded on block, and serial sagittal cryostat sections, cut at five levels, sectioned at 5 μm thickness, and mounted on glass slides. Each experiment was composed of six to ten sets of animals. A number of sections from each brain level were subjected to hematoxylin-eosin staining for the identification of morphological changes. For the histology and pathology diagnosis of brain tissue, the paraffin-embedded brain samples were stained by the dyes of 0.1% Mayers Hematoxylin and 0.5% Eosin (H&E Staining Kit, ab245880, Abcam, Cambridge, MA, USA), then dipped in distilled H2O, 50% EtOH, 70% EtOH, 95% EtOH, and 100% EtOH until the eosin stops streaking. The coverslips were mounted with Cytoseal XYL (Thermo Scientific, Waltham, MA, USA).

Immunohistochemistry serial sections that represented the parts of cerebellar medulla from control and GNMT^−/−^ mice were assayed simultaneously. The brain tissues were stained for the SMP30 (NBP1-80849, Novus Biologicals, Centennial, CO, USA), ADNP (NBP1-89236, Novus Biologicals, Centennial, CO, USA), RIPK1 (17519-1-AP, Proteintech Group Inc, Rosemont, IL, USA), Caspase 3 (19677-1-AP, Proteintech Group Inc, Rosemont, IL, USA), and Beta-actin (PA1-183, Thermo, Waltham, MA, USA). The results were captured by the Nikon microscope (Melville, NY, USA), and then processed by professional image software (Tucsen TCH-5.0, Fuzhou, Fujian, PRC).

### 2.4. Brain Proteome Research

The mouse brains were homogenized, and then the brain proteins were extracted by using the tissue protein extraction kit (RIPA, R0278, Merck). Protein samples (100 μL) were transferred into 1.5 mL Eppendorf tubes and incubated at 37 °C for three h after mixing with 25 μL of 10mM dithiothreitol (DTT, 15397, USB Corporation). The samples were reduced and alkylated in the dark at room temperature for 30 min after the addition of 25 μL of 55 mM iodoacetamide (IAA, RPN6302V, Amersham Biosciences, Little Chalfont, UK) in 25 mM ammonium bicarbonate. Approximately 10 μL of 0.1 μg/μL modified trypsin digestion buffer (Trypsin Gold, Mass Spectrometry Grade, V5280, Promega, Madison, WI, USA) in 25 mM ammonium bicarbonate was added to the protein samples, which were then incubated at 37 °C for at least 12 h in a water bath. Two microliters of formic acid were added to each sample before mass spectrometric analysis for protein identification.

Complex peptide mixtures were separated using RP-nano-HPLC-ESI-MS/MS. Each cycle of one full scan mass spectrum (m/z 400-2000) was followed by four data-dependent tandem mass spectra, with collision energy set at 35%. For protein identification, Mascot software was used to search the Swiss-Prot human protein sequence database. Proteins were initially annotated by similar searches using UniProtKB/Swiss-Prot (https://www.uniprot.org/) databases (accessed on 3 November 2020). The protein-protein interaction pathways were analyzed using Ingenuity Pathways Analysis (IPA).

### 2.5. Statistics Analysis

The statistical analysis of this study was carried out using SPSS v.20 (IBM Corp., Armonk, NY, U.S.A). The statistical method was analyzed by analysis of variance (ANOVA) and the LSD multiple comparison statistical method was used to test the differences between each group. Data presented is mean ± SD from at least hexaplicate measurements. The *p*-values were calculated using the unpaired two-sided Student’s t-test to compare groups, and *p* < 0.05 was considered statistically significant.

## 3. Results and Discussions

In this study, GNMT-KO mice were used as an animal model for early aging. The experimental results were divided into two parts. The protein markers mentioned in the literatures were adapted to confirm the aging or aging-related impairment of the brain by Western blotting and IHC. The IHC was performed for the whole brain. The full image of the microscope was taken using a low power lens, but this is unable to show the difference (Supplementary Figure S1). Thus, a section with higher magnification was selected to show more details, and the IHC staining of cerebellum stood out from the rest of the brain. Due to the limitation of the microscope, we only emphasized the cerebellum section for IHC even though the whole brain images were taken. In addition, the whole brain proteome and IPA pathway analyses were applied to explore the novel aging-related pathway. In addition, the whole brain proteome and IPA pathway analyses were applied to explore the novel aging-related pathway. The results of IHC in the cerebellum and proteome analysis in the whole brain were both confirmed that the GNMT-KO mice can be suitable as a rapid aging model and were related to the Gonadotropin-releasing hormone (GnRH) pathway.

### 3.1. H&E Stain

**Figure 1:**
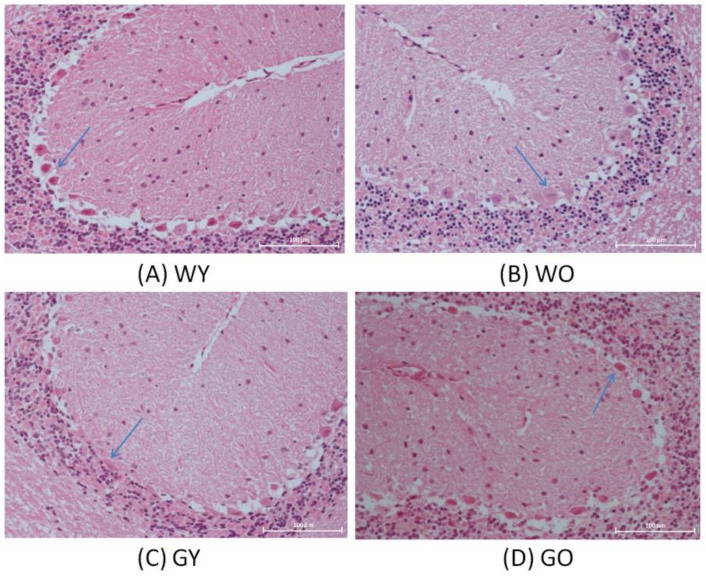
Hematoxylin and eosin staining histopathological examination of the brains from (**A**) WY, (**B**) WO, (**C**) GY, and (**D**) GO mice. The sizes and shapes of Purkinje cells located in the ganglion cell layer were different (200X). Abbreviations: young wild-type mice (WY); old wild-type mice (WO); young GNMT^−/−^ mice (GY); old GNMT^−/−^ mice (GO).

The results of H&E stained paraffin sections were shown in Figure 1. Among these four groups, the sizes and shapes of Purkinje cells located in the ganglion cell layer demonstrated the dissimilarity. The cerebellum is vital for the continuous smooth movements and may even be involved in cognitive processes and executive control. Purkinje cells (PCs) are nerve cells, which are relatively large in size and branched in shape. PCs are also involved in motor behavior. PCs are found in the cortex and are the sole output neurons of the cerebellum and can be a model to investigate synaptic specificity or synapse development/formation as well as understanding cerebellar function including cell-cell recognition and long-range axon guidance [15,16].The morphology of Purkinje cells in the young wild-type mice were complete and distinct compared with that of the WO mice having intact but being lightly stained; for the GY mice, Purkinje cells were compressed to the edge of the ganglion cell layer and the cell shape became narrow. For the GO mice, Purkinje cells were shrunken with darker staining.

The semi-quantitative analysis has been performed for well-known protein markers regarding neurodegeneration and aging include senescence marker protein 30 (SMP30), activity-dependent neuroprotective protein (ADNP), receptor-interacting protein 1 (RIPK1), and Caspase 3.

### 3.2. Senescence Marker Protein 30 (SMP30)

**Figure 2:**
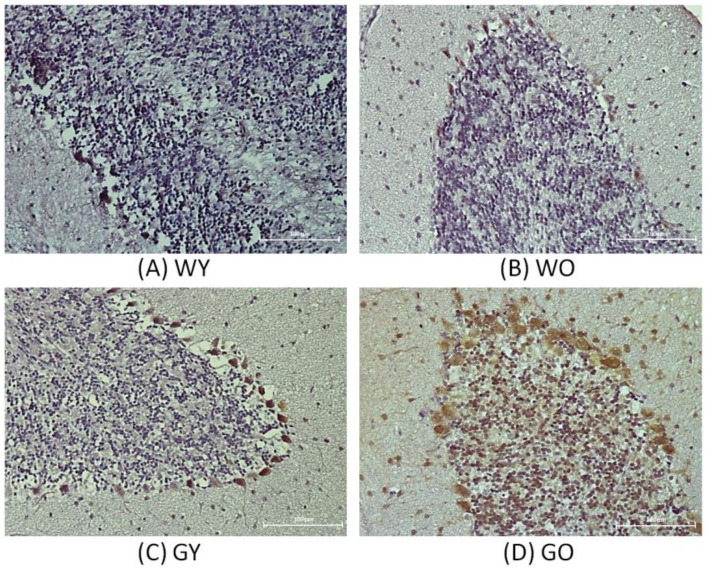
The results of immunohistochemistry staining of SMP30of the brain samples from (**A**) WY, (**B**) WO, (**C**) GY, and (**D**) GO mice. The cell density of the granular layer of the cerebellar medulla is denser and the staining are more concentrated in the samples of wild-type (Wt) mice (200X). Abbreviations: young wild-type mice (WY); old wild-type mice (WO); young GNMT^−/−^ mice (GY); old GNMT^−/−^ mice (GO).

**Figure 3:**
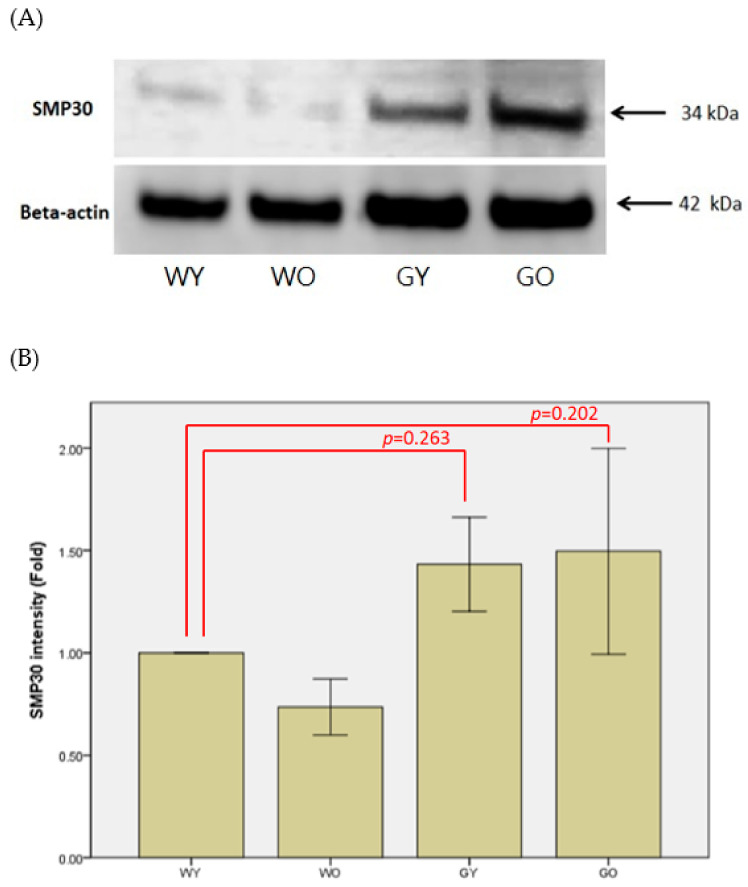
(**A**) Brain lysates from WY, WO, GY, and GO mice were collected, and levels of SMP30 were analyzed by Western blotting. (**B**) Beta-actin was used to normalize Western blot data (*n* = 8). Abbreviations: young wild-type mice (WY); old wild-type mice (WO); young GNMT^−/−^ mice (GY); old GNMT^-/-^ mice (GO).

Senescence marker protein 30, abbreviated as SMP30, is a type of calcitonin and regarded as an important aging marker. Figure 2 shows the results of immunohistochemistry staining of SMP30 in brain. The cell density of the granular layer of the cerebellar medulla is denser and the staining is more concentrated in the samples of Wt mice. For the samples from old wild-type (WO) mice and young GNMT^-/-^ mice (GY) mice, the cell densities gradually decrease where those of GO mice show the lowest cell density. Figure 2 shows that Purkinje cells in the sample of old GNMT^−/−^ mice (GO) mice expressed the highest level of SMP30 where the young wild-type (WY) mice samples expressed the lowest level of SMP30. Western blotting results show that the WY mice have lower expression of SMP30 compared with that of the GNMT-KO mice (Figure 3). In the samples of GNMT-KO mice, the GO mice expressed more SMP30 compared with that of the young ones. Due to the variation of GNMT-KO samples, there is no statistical significant difference among these samples. Nevertheless, the IHC shows that most of the SMP30 expression from the groups of WY, WO, and GY were in the granular layer where the intensity of the staining of GY was the highest among those groups. Interestingly, for the GO group, SMP30 expression was not only in the granular layer but also in cells from other areas. As a result, the expression level of SMP30 of GO group in WB was the highest.

Senescence marker protein 30 (SMP30), a calcium binding protein, is also known as an aging marker [17]. SMP30 is abundantly expressed in the liver and kidney. Its expression in rats decreases significantly with age [18]. When the gene of SMP30 was knocked out, the oxidative stress levels in the brain were elevated [19]. However, its expression in brain and cerebellum did not show a significant difference between 6 month and 21 month old rats [17]. In addition, when kainic acid (KA) was used as an agent to induce epilepsy causing brain and the nervous system damage, there was a loss of neurons in the hippocampus, and the SMP30 expression was increased. According to the immunohistochemical staining, the cell densities of the cerebellar medulla of the young Wt mice appear to be the densest, and those of old GNMT-KO mice are the most loose. Interestingly, Purkinje cells located in the ganglion layer were also stained by SMP30. Among them, the most highly expressed group is the old GNMT-KO mice, and the least expressed group is young Wt mice. Similar to Son’s study [17], there was no significant expression change along with aging. Interestingly, the expression was elevated in GNMT-KO mice, but the expression level difference between the old and young GNMT-KO mice was inconclusive. Nevertheless, the elevated expression in GNMT-KO mice may indicate neuron damage.

### 3.3. Activity-Dependent Neuroprotector (ADNP)

**Figure 4:**
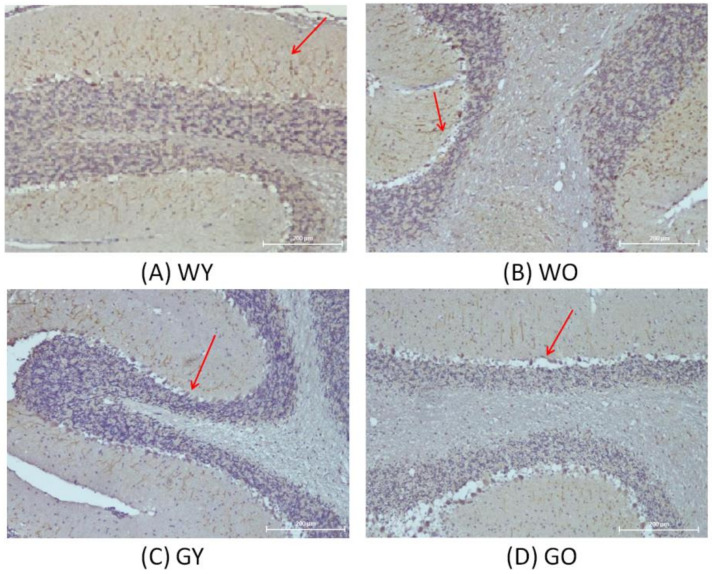
Immunohistochemistry (IHC) of activity-dependent neuroprotective protein (ADNP)using mouse brain samples from (**A**) WY, (**B**) WO, (**C**) GY, and (**D**) GO mice. The nerve fibers, located in the molecular layer of the cerebellar medulla, are more densely distributed and have a longer length in WY mice (100X). Abbreviations: young wild-type mice (WY); old wild-type mice (WO); young GNMT^−/−^ mice (GY); old GNMT^−/−^ mice (GO).

**Figure 5:**
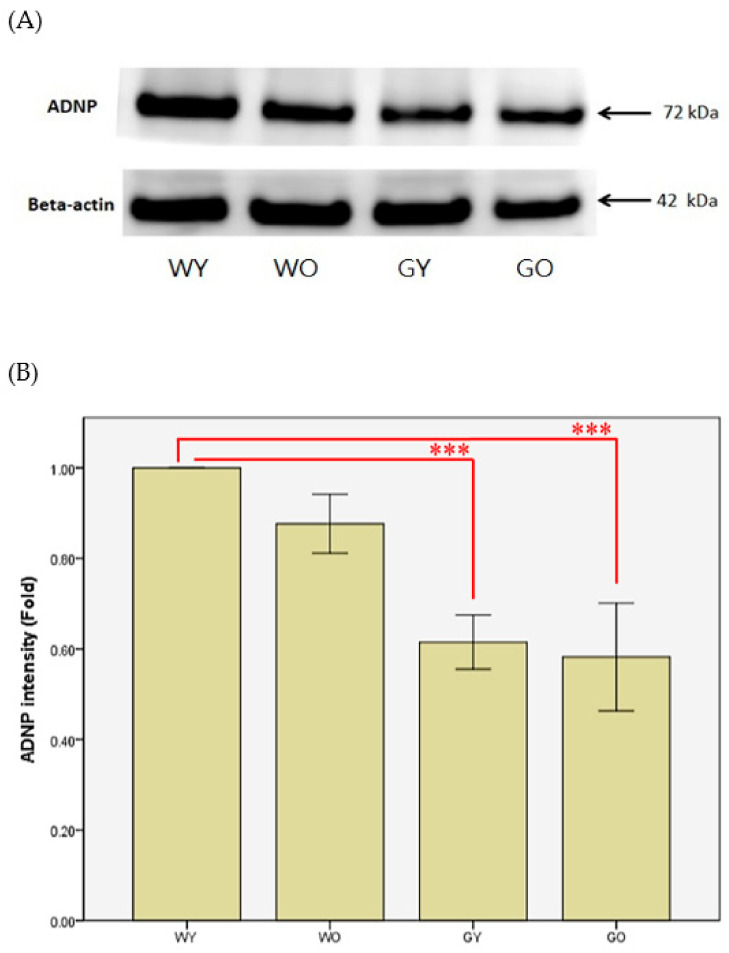
(**A**) Brain lysates from WY, WO, GY, and GO mice were collected, and levels of ADNP were analyzed by Western blotting. (**B**) Beta-actin was used to normalize Western blot data (*n* = 8, *** *p* < 0.001). Abbreviations: young wild-type mice (WY); old wild-type mice (WO); young GNMT^−/−^ mice (GY); old GNMT^−/−^ mice (GO).

Activity-dependent neuroprotective protein is abbreviated as ADNP. Figure 4 shows that the nerve fibers, located in the molecular layer of the cerebellar medulla, have denser distribution and a longer length in WY mice. As for WO and GY mice, the distribution of nerve fibers is very loose, but is the loosest in GO mice. It shows that WY mice had less localized vacuolation at the edge of the cerebellar granular layer while GO mice had the largest area having vacuolation among all four groups. Figure 5 shows that the expression levels of ADNP in Wt mice are greater than those of the GNMT-KO mice groups. The differences between WY and GNMT-KO are significant (*p* < 0.001).

The mRNA expression of ADNP (activity-dependent neuroprotective protein) is regulated by the neuroprotective peptide vasoactive intestinal peptide (VIP) and is also considered as one of the mediators of VIP-induced neuroprotection [20]. The active form of ADNP was identified as NAP (NAPVSIPQ, Asn-Ala-Pro-Val-Ser-Ile-Pro-Gln) [21]. The ADNP gene can be found and expressed in a variety of tissues and cells, including lymphocytes, fibroblasts, and nervous systems. In brains of human and mouse, the main expression areas of ADNP are in the cerebellum, hippocampus, and cerebral cortex [22,23]. From the perspective of the development of organisms, experiments have pointed out that mutant mice lacking the ADNP gene, in the early embryonic period, can be observed to have obvious growth retardation in the uterus, and even inhibition of brain formation [24]. It is also shown that ADNP is an essential protein for brain function, and plays an important role in the ability to have normal cognitive function [24,25]. In addition, NAP has been shown to have neuroprotective activity against Alzheimer’s disease using the in vitro system [26]. Thus, it is not surprising to find that such protein expression was much lower in GNMT-KO mice.

### 3.4. Receptor-Interacting Protein 1 (RIPK1)

**Figure 6:**
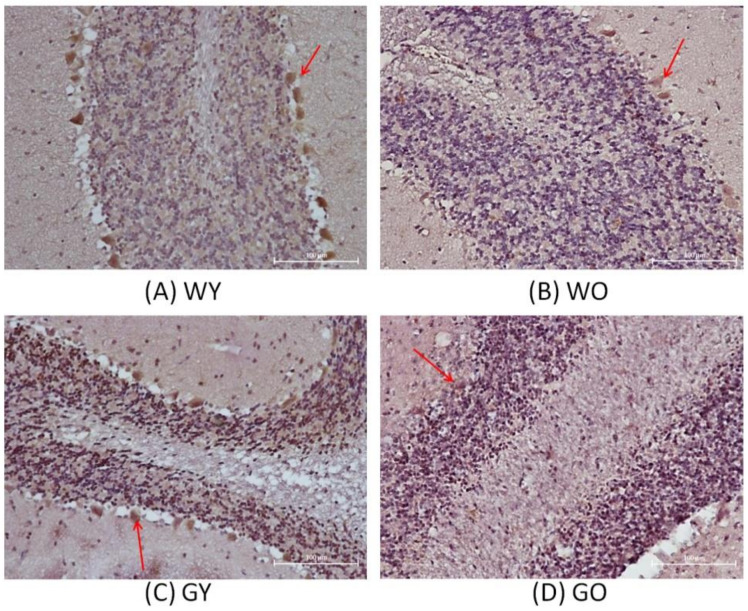
Immunohistochemistry (IHC) of RIPK1 using mouse brain samples from (**A**) WY, (**B**) WO, (**C**) GY, and (**D**) GO mice. The cell morphology of Purkinje cells in WY is intact, which is not the case for the WO and GY group. The cell morphology of Purkinje cells in GO is incomplete and fragmented (200X). Abbreviations: young wild-type mice (WY); old wild-type mice (WO); young GNMT^−/−^ mice (GY); old GNMT^−/−^ mice (GO).

**Figure 7:**
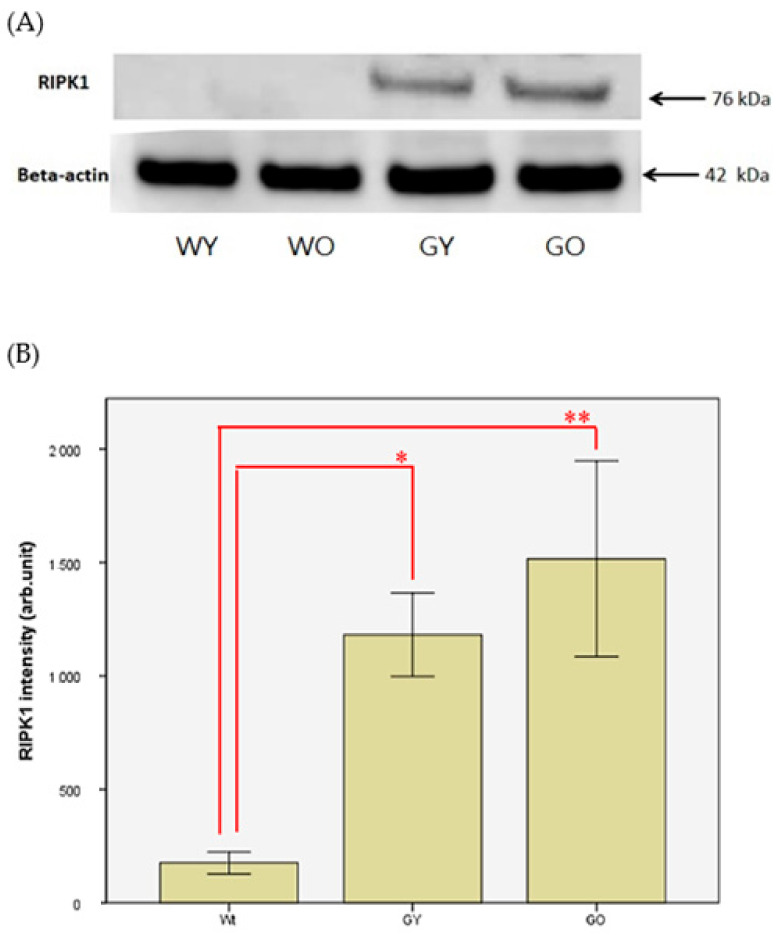
(**A**) Brain lysates from Wt, GY, and GO mice were collected, and levels of RIPK1 were analyzed by Western blotting. The RIPK1 expression was down-regulated or non-detectable in some WY and WO mice; thus, the WY and WO mice were combined into one group as Wt. (**B**) Beta-actin was used to normalize Western blot data (*n* = 8, * *p* < 0.05 and ** *p* < 0.005). Abbreviations: wild-type mice (Wt); young GNMT^−/−^ mice (GY); old GNMT^−/−^ mice (GO).

Receptor-interacting protein kinase 1, also known as RIPK1, is responsible for regulating cell apoptosis and necrotic death. Figure 6 shows the differences of cell morphology and sizes of Purkinje cells. The cell morphology of Purkinje cells in WY was intact, which was not the case for the WO and GY groups. The cell morphology of Purkinje cells in GO was incomplete and fragmented. Figure 7 shows the representative Western blotting results of RIPK1 from brain lysates, which were almost not detectable in the sample of Wt. On the other hand, the protein expression in both GY and GO mice is much higher than that of Wt. Due to the expression of RIPK1 being nearly not detectable in Wt mice, the data of WY and WO mice were combined into one group as Wt., and the unit of y-axis used arb. unit instead of Fold. The differences are significant (*n* = 8, *p* < 0.05 and 0.005). After normalization using β-actin, the concentrations of RIPK1 were almost undetectable in Wt mice, which were 1/6 to 1/8 to the levels detected in GY and GO mice (Supplementary Materials Figure S2).

Receptor-interacting protein kinase 1 (RIPK1) is considered to be an important molecule responsible for the regulation of inflammation, apoptosis, and necroptosis [27]. It has also been indicated that the inhibition of RIPK1 can alleviate the neuroinflammatory symptoms caused by cerebral ischemia and protect the brain from acute and chronic brain injury [28]. It has been shown that RIPK1 can regulate the related neuroinflammation of Alzheimer’s disease (AD) and inhibiting the activation of RIPK1 can reduce inflammation and cognitive impairment caused by Alzheimer’s disease [29]. Our immunohistochemistry staining showed that the glial cells located in the white matter of the cerebellum were stained. Some studies showed that RIPK1 is highly expressed by glial cells in the brain with AD [29]. Our experimental results demonstrated that RIPK1 expression in glial cells of the old GNMT-KO group was the highest among all groups. In addition, our Western blotting results showed no differential expression of RIPK1 between the young and old Wt mice. On the other hand, RIPK1 was highly expressed in both young and old GNMT-KO groups. This may be due to RIPK1 being mainly responsible for mediating necrotic death, which is relatively less important in Wt group. Since GNMT is involved in cellular defense against DNA damage and plays an important role in normal neurons, it is not surprising to have such an observation [30]. It is likely that during aging, the apopotic activity gradually decreases [31]. Under such an insufficient apoptosis condition, this may activate the necrotic death response, and RIPK1 is an important mediating protease. However, this can cause inflammation, which may be related to the pathogenesis of central nervous system-related diseases. The process of necrotic death may produce harmful mediators affecting the degeneration of neurons. However, in general, necrotic death does not participate in the development of normal adult individuals. In some chronic neurodegenerative diseases, such as Alzheimer’s disease (AD), Parkinson’s disease (PD), and multiple sclerosis (MS), related ligands may be activated and further developed into necrotic cell death with induced inflammation.

### 3.5. Caspase 3

**Figure 8:**
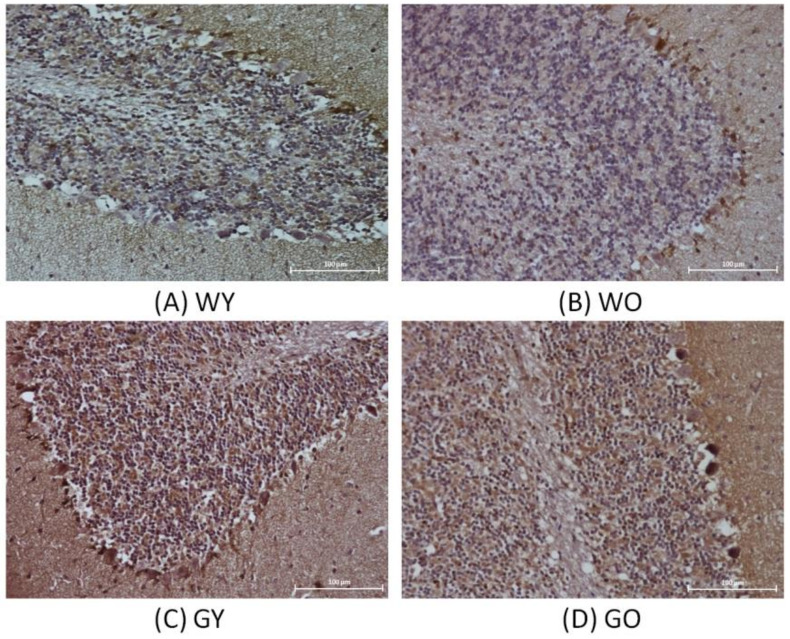
Immunohistochemistry (IHC) of Caspase 3 using mouse brain samples from (**A**) WY, (**B**) WO, (**C**) GY, and (**D**) GO mice. The staining is located in the granular layer of the cerebellar medulla (200X). Abbreviations: young wild-type mice (WY); old wild-type mice (WO); young GNMT^−/−^ mice (GY); old GNMT^−/−^ mice (GO).

**Figure 9:**
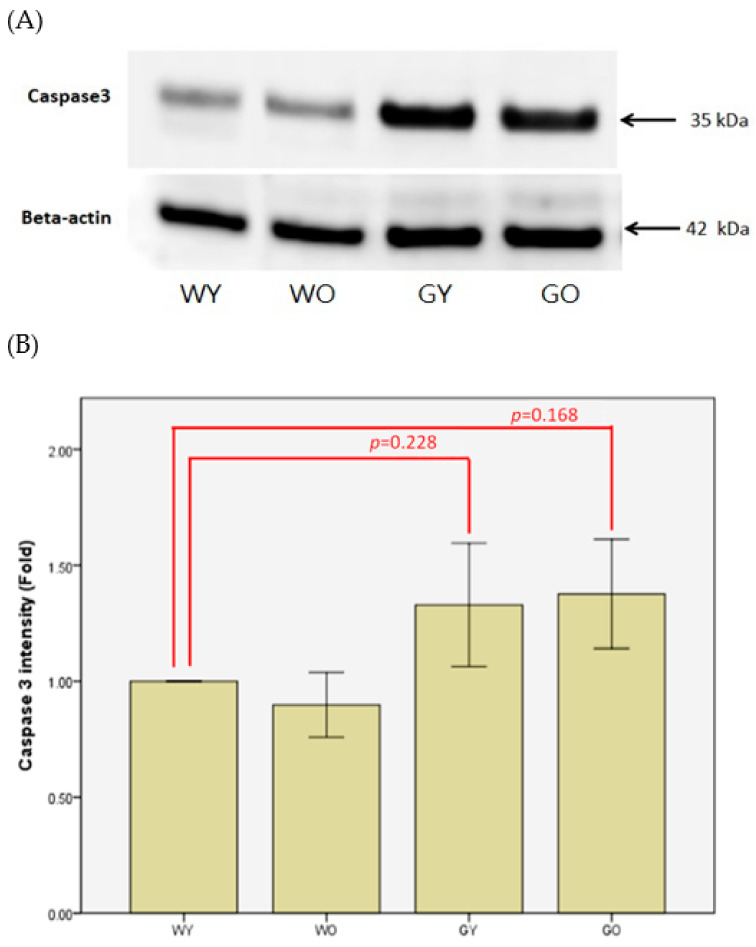
(**A**) Brain lysates from WY, WO, GY, and GO mice were collected, and levels of Caspase 3 were analyzed by Western blotting. (**B**) Beta-actin was used to normalize Western blot data (*n* = 8). Abbreviations: young wild-type mice (WY); old wild-type mice (WO); young GNMT^−/−^ mice (GY); old GNMT^−/−^ mice (GO).

Caspase 3 is a protease with the ability to mediate cell death. The IHC staining shows that the staining is located in the granular layer of the cerebellar medulla, and the protein expression in Wt group was much lower than those of GNMT^−/−^ groups (Figure 8). The protein expression detected by immunoblotting also shows similar results (Figure 9).

Caspase 3 is a caspase protease and also named as a death-mediated protease. It is an enzyme functioning in differentiation, remodeling, and mediating death. Caspase 3 has been discussed regarding neurodegenerative diseases and aging. The mechanism of apoptosis induced by Caspase 3 has also been extensively studied in neurons in the cerebral cortex, hippocampus, and cerebellum [32]. Louneva and coworkers showed that mice inherently lacking caspase 3 gene had neuronal apoptosis during development [33]. Although the connection of neurosenescence or cell death related to Caspase 3 is still unclear, it can be confirmed that Caspase 3 is a major receptor of neuronal apoptosis. In the process of aging, the death of neurons in the cerebral cortex and hippocampus is inevitable. However, it has been reported that, in the normal aging process, the death of neurons is restricted and the expression of Caspase 3 is not increased [34]. Our IHC results also show that Caspase 3 was expressed mostly in Purkinje cells in the cerebellar medulla. This is consistent with other reports that Caspase 3 is mostly expressed in the layers of Purkinje cells and granular cells. Since Caspase 3 was heavily stained in the brain samples from the GNMT-KO group, there may be more cell death than those of other groups. In addition, the increased expression of Caspase 3 was not observed in the old Wt mice; this also agrees with Shimohama’s study [34]. Although apoptosis and necrosis regulate cell death through different mechanisms and caspases are responsible for participating in neural development and mediating cell apoptosis, in the case of insufficient apoptotic response, caspases will activate necrosis [35]. Caspases are usually regarded as a protease capable of catalytic activity. It can mediate the apoptotic response of vertebrate neurons, and can be activated through internal cellular pathways starting from DNA damage where the downstream effectors are Caspase 3 and Caspase 9. Once this process is started, the caspases will cleave and release substances to target the cell death [34]. In the early neuron development process, the generation of apoptotic response contributes to the establishment and formation of the central nervous system. Apoptosis can remove excess neurons and improve the connection between cells. Therefore, cells are highly sensitive to the apoptotic response in the early stage of central nervous system development, but become insensitive or even develop resistance after maturity. Our results showed that the protein expression level of Caspase 3 in old Wt mice was slightly decreased, but such expression in GNMT-KO mice was increased. The old Wt mice may have other replaced responses for apoptosis where the GNMT-KO mice may have some severe nerve damage.

### 3.6. Biomarkers in Aged Mice- Proteome Database Search

After data analysis using Mascot analysis software, 198 proteins were identified from the young wild-type mice (WY) and were applied as the control. These control proteins were then compared with 184 proteins from the old wild-type mice (WO), 175 proteins from the young GNMT^−/−^ mice (GY), and 171 proteins from the old GNMT^−/−^ mice (GO). The differential expressed proteins were further discussed.

**Figure 10:**
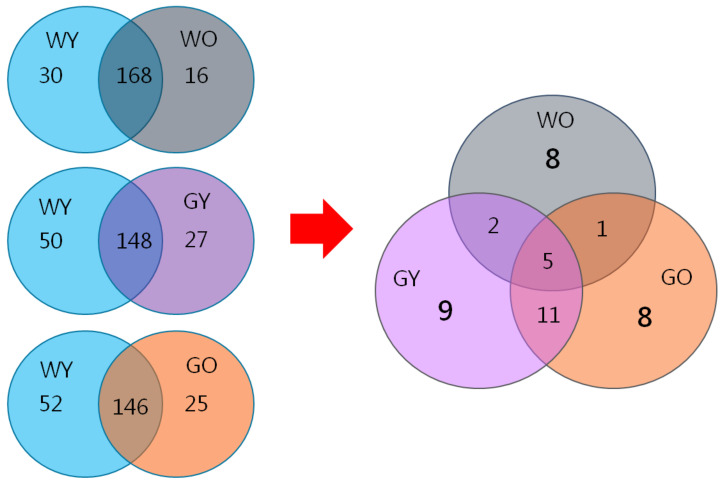
The Mascot results indicate that 198 proteins were unique for the groups of WY mice. Compared with the WY mice, 16, 27, and 25 proteins were unique for the groups of WO, GY, and GO mice, respectively. After deducting the proteins found from the WY mice and common proteins, there are only eight, nine, and eight unique proteins in the groups of WO, GY, and GO mice. Abbreviations: young wild-type mice (WY); old wild-type mice (WO); young GNMT^−/−^ mice (GY); old GNMT^−/−^ mice (GO).

After deleting the repetitive sequences, there were 16, 27, and 25 unique proteins identified in the groups of WO, GY, and GO, respectively (Figure 10). After re-arranging proteins from these groups and removing the overlapped ones, the WO, the GY, and the GO groups showed eight, nine, and eight distinctive proteins, respectively. Their characteristics and protein functions are listed in Table 1. These proteins are found to be related to aging and neurology.

Among proteins solely identified in the group of WO mice (Table 1), Excitatory amino acid transporter 2, Calcium/calmodulin-dependent protein kinase type II subunit alpha, and Cu -Zn Superoxide dismutase are related to neuropathology. Excitatory amino acid transporter 2 (EAAT2) is related to neurodegenerative diseases. EAAT2 is a transporter that expresses glutamine (Glutamate) mainly in astrocytes, and the role of glutamine is responsible for regulating nerve excitatory transmission signals in the brain. Studies have shown that the dysfunction of EAAT2 is related to the accumulation of glutamine and may be related to neurodegenerative diseases, such as Alzheimer’s disease (AD) and Huntington’s disease (a.k.a. Huntington’s chorea) and amyotrophic lateral sclerosis (Amyotrophic lateral sclerosis) [36,37,38]. Calcium/calmodulin-dependent protein kinase type II subunit alpha is associated with age-related intracellular redox. Changes in the activity of Ca^2+^ /calmodulin-dependent protein kinase II (CaMKII) can be found in the aged hippocampus, which represents its oxidation changes in the relationship with neurosynaptic plasticity [39,40]. The Cu-Zn superoxide dismutase (SOD) is related to the regulation of oxidative damage. Related studies have shown that excessive SOD in the body may generate oxidation-derived free radicals causing acceleration of age and aging-related neuropathology, such as peroxidative brain damage. Therefore, the changes of SOD levels may be one of the important factors [41,42]. These may be healthy aging markers.

Among proteins identified only in the group of GY mice, 4-aminobutyrate aminotransferase, Limbic system-associated membrane protein, Sodium- and chloride-dependent GABA transporter 3, and ProSAAS are related to function of neurons. Abat, 4-aminobutyrate aminotransferase may be related with neurological damage symptoms including cognitive impairment and epilepsy if the expression of Abat is deficient. In addition, the Abat-related pathway has been targeted to develop therapeutic drugs for neurological diseases [43]. Limbic system-associated membrane protein (LSAMP) is related to the lens protein in aging patients [44]; it was also found that immobilized LSAMP substrates can inhibit neurite outgrowth in neurons of dorsal root ganglion cells [45]. Sodium- and chloride-dependent GABA transporter 3 is related to the expression of aging-related brain genes [46]. ProSAAS is highly expressed in brain neurons and neuroendocrine tissues. The function of proSAAS includes the inhibition of endogenous prohormone convertases 1, which has catalytic activities of releasing hormones or neuropeptides [47]. In addition, since proSAA Simmunal responses can be observed in the brains of patients with dementia syndromes (such as Alzheimer’s disease and Parkinson’s disease), this renders proSAAS as a potential therapeutic target in neurodegenerative diseases [48,49,50]. These may be markers for mild neurological disorders.

Combined with aging and GNMT deficiency effects, GO mice may demonstrate markers for more severe neurological disorders. Among proteins exclusively identified in the group of GO mice, eight proteins are related with aging (Table 1). Among those, serum albumin is interesting. Skillbäck and coworkers compared the CSF (cerebral spinal fliud)/serum albumin ratios from dementia patients to those of healthy control groups and showed that BBB (blood-brain barrier) leakage is common in dementias [51]. Since the subcellular location of albumin is in the extracellular region, the elevated level of albumin may be due to the leakage. In addition, the Rho GDP dissociation inhibitor 1, which may be related to neurogenesis, neuroprotection, or synaptic strength, also plays a role in the hippocampus. Since Rho GTPases’ involvement in neurodegenerative disorders has been implicated and several intrinsic signaling pathways and neuronal process regulations, such as neuronal migration and polarization, axon guidance and dendrite formation, as well as synaptic organization and plasticity [52,53] are involved, the elevation of rho GDP dissociation inhibitor 1 may have a negative impact. These may be markers for aggravated neurological disorders with aging.

### 3.7. Enriched Biological Pathways by IPA Analysis

The proteomic experimental results were examined by IPA analysis. The relationships among these differential expressed proteins were examined to determine the canonical pathways and biological networks involved. The most significant canonical pathways were presented in Table 2. Our analysis revealed these canonical pathways were connected with GnRH Signaling for old mice.

IPA system, also known as biological path analysis software and database, is an advanced bioinformatic tool using internally constructed databases to analyze the expression patterns of related proteins and genes [54]. The establishment of the Ingenuity knowledge base is a collection of large-scale observation and various experimental results [55]. This system targets genes, compounds, and microRNAs to discuss their biological-related functions among nearly 5 million findings in this database. IPA was adapted to investigate the Canonical Pathway Analysis. Table 2 list the top 23 pathways analyzed from the groups of old mice. Interestingly, the GnRH signaling showed importance in both old mice groups.

GnRH, also known as Luteinising-hormone releasing hormone, is a poly-peptide substance located outside the hypothalamus-pituitary-gonadal (Hypothalamic-pituitary-gonadal, HPG) axis. It also regulates reproductive activities. GnRH is secreted periodically from neurons in the hypothalamus into the portal circulation. By binding to and activating the GnRH receptors of the pituitary gonadotropin cells, the receptors synthesize gonadotropins, such as Luteinizing Hormone (LH) and Follicle-stimulating hormone (FSH). These gonadotropins stimulate cell proliferation and secrete gonadal steroids [56,57,58]. Regarding the generation of GnRH neurons, it has been reported that GnRH was confirmed to originate from the central nervous system (CNS) during the embryonic period [59]. Newly generated GnRH neurons move along with the duct formed by the olfactory bulb to the medial preoptic area of the hypothalamus. Once it reaches this area, neurons will expand the axon, so that gonadotropins can be secreted to the portal system of the pituitary in a coordinated manner. In this way, GnRH neurons can accurately monitor and receive feedback from various sensory and nervous systems. GnRH not only acts on the pituitary gland, but also in glands of breast, placenta, prostate, central, and peripheral nervous systems. GnRH may target at non-pituitary peripheral subjects. Ligands and receptors of GnRH in the brain may be involved in learning, memory, or feeding behavior [56]. It was also suggested that the pituitary response to GnRH was attenuated with aging [60]. Thus, GnRH may have important roles in aging. In addition, IPA showed that the effect of GnRH in Wt mice was greater. This may be due to the heavy involvement of GnRH in the reproductive system. Interestingly, it has been shown that agonistic therapy of GnRH played a beneficial role in neurodegenerative disease in a small cohort study [56].

**Figure 11:**
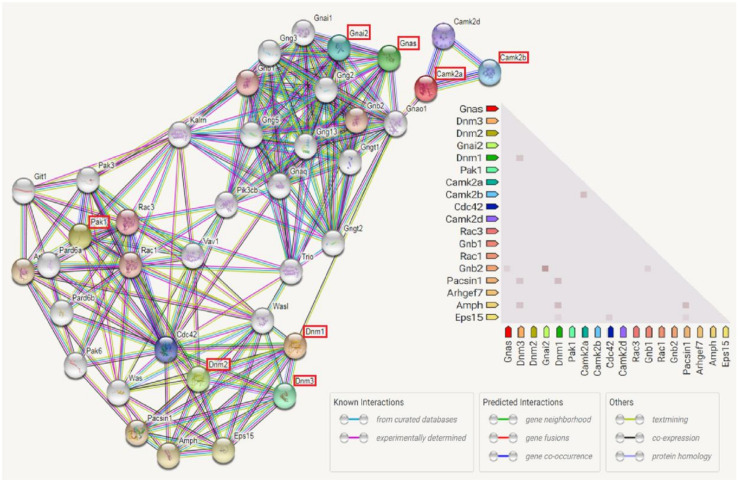
The protein-protein interaction pathways were illustrated. Eight proteins identified from the Ingenuity Pathway Analysis (IPA) were PAK1, DNM1, DNM2, DNM3, GNAI2, GNAS, CAMK2A, and CAMK2B, which were inter-connected with other proteins.

By using IPA pathway analysis, several proteins in the GnRH signaling pathway were involved in our study, such as Serine/threonine-protein kinase (PAK1), Dynamin (DNM1, DNM2, DNM3), Guanine nucleotide-binding protein G (GNAI2, GNAS), and Calcium/calmodulin-dependent protein kinase (CAMK2A, CAMK2B). Although this is not evidenced by our wet lab work, the literature-based evidences were results from experiments such as Western Blot, immunohistochemistry, real time PCR, proteomics, etc. The protein-protein interactions among those eight proteins are shown in Figure 11 where PAK1, DNM1, DNM2, DNM3, GNAI2, GNAS, CAMK2A, and CAMK2B had complex interactions with other proteins.

Neurodegenerative diseases are one of the leading causes of death and disability worldwide. PAK1 is involved in intracellular signaling pathways downstream of in-tegrins and receptor-type kinases and plays an important role in cytoskeleton dynam-ics, cell adhesion, migration, proliferation, apoptosis, mitosis, and vesicle-mediated transport processes as well as involving in the pathophysiology of neurodegenerative diseases, such as Alzheimer’s Disease (AD) and cerebral ischemia [61]. PAK1 expres-sion was altered with aging. This reduction in level and activity were mainly found in the hippocampus resulting decreased cell viability and increased cell death induced by oxidative stress [62].

Dynamin (DNM) proteins are known as synaptic proteins, which are the keys in the presynaptic regulation of endocytosis. DNMs ensure the survival of postmitotic neurons, including Purkinje cells, by suppressing oxidative damage in addition to being required for normal cerebellum development. It also facilitates developmentally regulated apoptosis during neural tube formation [63]. Additionally, the dynamin 1 levels in the brain were significantly lower in the aged group, especially in the Alzheimer’s patients [64].

Guanine nucleotide-binding proteins (GNAI2 and GNAS) are involved as modulators or transducers in various transmembrane signaling systems including activation of adenylyl cyclases, which results in increased levels of the signaling molecule, cAMP. During the aging process, this age-related alteration in the central nervous system (CNS), particularly in age-vulnerable regions (hippocampus, striatum, frontal cortex, and substantia nigra) is easily influenced, causing dysfunction and leading the development of neuropsychiatric disease in the later years of life. This includes brain function decline, neuroregeneration impairement, and increased vulnerability to neuropathologies, such as Parkinson and Alzheimer’s diseases [65,66].

Calcium/calmodulin-dependent protein kinase (CAMK2) functions autonomously after Ca^2+^/calmodulin-binding and autophosphorylation. It is involved in dendritic spine and synapse formation, neuronal plasticity, and regulation of sarcoplasmic reticulum Ca^2+^ transport in skeletal muscle [67]. In neurons, it was found to play an essential structural role in the reorganization of the actin cytoskeleton during plasticity by binding and bundling actin filaments in a kinase-independent manner. Under normal physiological conditions, the expression levels of CAMK2, both mRNA and protein, were decreased significantly in the cerebral cortex along with aging [68]. However, the expression of CAMK2 significantly increased in the cerebral cortex and hippocampus from samples of the Alzheimer’s disease animal model [40]. In addition, it was found that an aberrant CAMK2 activation to promoting neurodegeneration was caused by phosphorylated tau at the Alzheimer’s disease-associated sites, Ser262/356. CAMK2 dysregulation may be a modulator of toxicity in Alzheimer’s disease [69].

### 3.8. GNMT and Aging

Our goal is to identify neurogenesis related aging biomarkers where a mouse model of GNMT gene knockout was used. Due to the lack of this gene, the content of methionine and SAM in mice increased about 3 and 70 fold compared with those of Wt mice, respectively [70]. When GNMT is not expressed in mice, it also causes higher SAM level in hippocampal gyrus. Improper SAM concentrations may reduce neuron activities, damage nerves, and a decline in cognitive ability as well as disabilities in memory and spatial learning [71]. In addition, the deletion of GNMT may cause excessive accumulation of homocysteine (Hcy) where hyperhomocysteinemia (HHcy) has been proven to be one of the risk factors for the neurological diseases or neurodegenerative disorders [72]. Many neurodegenerative diseases, such as Alzheimer’s disease and dementia, are related to alterations in SAMe/SAH ratio. This also represents the change in the ratio of SAM to SAH is potentially related to cognitive ability. Therefore, in our experiments, we used known protein markers to confirm the aging and damage of the brains. Proteins identified with aging in brain indicate that the GnRH pathway may play an important role in the aging process.

## 4. Conclusions

In this study, we used GNMT^−/−^ mice as the experimental model for the research on aging. From the literature, it indicates that SMP30, ADNP, RIPK1, and Caspase 3 are significantly associated with neuro disorders and aging in brain. The concentrations of expectant proteins in this study were also determined by Western blotting and IHC. The exact role of GNMT in neurogenesis may need to be elucidated, but its deficiency plays a role in neurodegeneration. In summary, this study demonstrates that the GnRH pathway provided here may shed light on the molecular mechanisms for aging. The proteins meaningful for general aging and non-aging related neurodegenation, which were identified by the proteomic approaches, also confirmed that aging may regulate brain and nerve damage-related proteins. These markers will contribute to better understanding of aging and may be served for drug discovery as therapeutic or prevention targets.

## Figures and Tables

**Table 1:** The proteins identified with higher confidence levels and summary of protein function corresponding to aging in this study.

Swiss-Port TrEMBL Accession Number	Protein Name	MW	Score	Match Queries	p*I*	Sequence Coverage (%)	Matched Peptide	Subcellular Location	Protein Function
**WO mice**									
P43006	Excitatory amino acid transporter 2	61,990	43	4	6.24	8	MASTEGANNMPK.Q+Deamidated (NQ) K.KNDEVSSLDAFLDLIR.N R.CLEDNLGIDK.R +Carboxymethyl (C) K.SELDTIDSQHR.M	Cell membrane; Multi-pass membrane protein	Functions as a symporter that transports one amino acid molecule together with two or three Na+ ions and one proton, in parallel with the counter-transport of one K+ ion.
P15864	Histone H1.2	21,254	100	5	11	18	K.ERSGVSLAALK.K +Phospho (ST) R.SGVSLAALKK.A K.KALAAAGYDVEKNNSR.I +Deamidated (NQ) K.ALAAAGYDVEKNNSR.I K.GTGASGSFKLNK.K +Phospho (ST)	Nucleus; Chromosome	Histones H1 are necessary for the condensation of nucleosome chains into higher-order structured fibers. Acts also as a regulator of individual gene transcription through chromatin remodeling, nucleosome spacing and DNA methylation.
P11798	Calcium/calmodulin-dependent protein kinase type II subunit alpha	54,081	31	2	6.61	7	R.KQEIIKVTEQLIEAISNGDFESYTK.M+2 Phospho (ST) R.FYFENLWSR.N	Cytoplasm; Synapse	Calcium/calmodulin-dependent protein kinase that functions autonomously after Ca^2^/calmodulin-binding and autophosphorylation, and is involved in synaptic plasticity, neurotransmitter release and long-term potentiation.
Q923T9	Calcium/calmodulin-dependent protein kinase type II subunit gamma	59,569	55	4	7.32	9	MATTATCTR.F +Carboxymethyl (C); 2 Phospho (ST) K.IINTKKLSAR.D +Deamidated (NQ); 2 Phospho (ST) R.DLKPENLLLASK.C K.AGAYDFPSPEWDTVTPEAK.N	Sarcoplasmic reticulum membrane; Peripheral membrane protein; Cytoplasmic side	Calcium/calmodulin-dependent protein kinase in the central nervous system, it is involved in the regulation of neurite formation and arborization. It may participate in the promotion of dendritic spine and synapse formation and maintenance of synaptic plasticity which enables long-term potentiation (LTP) and hippocampus-dependent learning.
Q8C650	Septin-10	52,388	40	2	6.17	4	R.AQTYELQESNVR.L +3 Deamidated (NQ); Phospho (ST) K.VNIIPLIAK.A	Cytoskeleton; Cytoplasm	May play a role in cytokinesis.
P08228	Superoxide dismutase [Cu-Zn]	15,933	69	2	6.02	16	K.GDGPVQGTIHFEQK.A R.HVGDLGNVTAGK.D	Nucleus; Cytoplasm	Destroys radicals which are normally produced within the cells and which are toxic to biological systems.
Q62277	Synaptophysin	34,002	37	3	4.82	7	K.MATDPENIIK.E K.MATDPENIIK.E +Deamidated (NQ); Oxidation (M); Phospho (ST) K.EMPMCRQTGNTCK.E +Carboxymethyl (C); Deamidated (NQ)	Synaptic vesicle Membrane; Membrane protein; Synaptosome	Involved in the regulation of short-term and long-term synaptic plasticity.
Q9R0P9	Ubiquitin carboxyl-terminal hydrolase isozyme L1	24,822	71	4	5.14	24	MQLKPMEINPEMLNK.V+Deamidated (NQ); 2 Oxidation (M) K.LEFEDGSVLK.Q K.QFLSETEK.L R.MPFPVNHGASSEDSLLQDAAK.V	Endoplasmic reticulum Membrane; Cytoplasm	Ubiquitin-protein hydrolase was bound to free monoubiquitin and may prevent its degradation in lysosomes. The homodimer may have ATP-independent ubiquitin ligase activity.
**GY mice**									
P61922	4-aminobutyrate aminotransferase, mitochondrial	56,416	27	1	8.35	3	K.AIHKIDIPSFDWPIAPFPR.L	Mitochondrion matrix	Catalyzes the conversion of gamma-aminobutyrate and L-beta-aminoisobutyrate to succinate semialdehyde and methylmalonate semialdehyde, respectively. Can also convert delta-aminovalerate and beta-alanine.
P08752	Guanine nucleotide-binding protein G(i) subunit alpha-2	40,463	41	3	5.28	10	K.LLLLGAGESGK.S K.STIVKQMK.I +Oxidation (M); Phospho (ST) R.QYRAVVYSNTIQSIMAIVK.A +2 Deamidated (NQ); Oxidation (M)	Plasma membrane; Cell membrane; Cytoskeleton	The G(i) proteins are involved in hormonal regulation of adenylate cyclase: they inhibit the cyclase in response to beta-adrenergic stimuli. May play a role in cell division.
Q8BLK3	Limbic system-associated membrane protein	38,063	25	5	6.21	18	R.GTDNITVR.Q +Phospho (ST) R.HALEYSLR.I K.AANEVSSADVK.Q +Deamidated (NQ) K.AANEVSSADVKQVK.V K.VTVNYPPTITESKSNEATTGRQASLK.C +2 Deamidated (NQ); 6 Phospho (ST); Phospho (Y)	Cell membrane	Mediates selective neuronal growth and axon targeting. Contributes to the guidance of developing axons and remodeling of mature circuits in the limbic system. Essential for normal growth of the hyppocampal mossy fiber projection.
P35486	Pyruvate dehydrogenase E1 component subunit alpha, somatic form, mitochondrial	43,204	45	2	8.49	4	K.ADQLYK.Q +Deamidated (NQ); Phospho (Y) R.SKSDPIMLLKDR.M	Mitochondrion matrix	The pyruvate dehydrogenase complex catalyzes the overall conversion of pyruvate to acetyl-CoA and CO2, and thereby links the glycolytic pathway to the tricarboxylic cycle.
Q8CGM2	Retinitis pigmentosa 1-like 1 protein	199,562	26	4	6.06	3	K.VCMNEDGSLSVEMK.V +Carboxymethyl (C); 2 Phospho (ST) K.VRLEESPKYQEMLR.L +Oxidation (M) R.MLSQEDLGTVQGADEK.Q K.GEDEGSAGSLACTQVGGK.V +3 Phospho (ST)	Cytoskeleton	Plays a role in the organization of outer segment of rod and cone photoreceptors.
P31650	Sodium- and chloride-dependent GABA transporter 3	69,914	24	1	6.66	2	K.VKGDGTISAITEKETHF.	Membrane	Terminates the action of GABA by its high affinity sodium-dependent reuptake into presynaptic terminals. Can also transport beta-alanine and taurine.
Q9CWF2	Tubulin beta-2B chain	49,921	362	14	4.78	41	K.FWEVISDEHGIDPTGSYHGDSDLQLER.I R.INVYYNEATGNKYVPR.A R.AILVDLEPGTMDSVR.S R.AILVDLEPGTMDSVR.S +Oxidation (M) R.SGPFGQIFRPDNFVFGQSGAGNNWAK.G K.GHYTEGAELVDSVLDVVR.K K.GHYTEGAELVDSVLDVVRK.E K.LAVNMVPFPR.L R.LHFFMPGFAPLTSR.G R.LHFFMPGFAPLTSR.G +Oxidation (M) R.ALTVPELTQQMFDSK.N R.YLTVAAIFR.G K.NSSYFVEWIPNNVK.T K.MSATFIGNSTAIQELFKR.I	Cytoskeleton	Tubulin plays a critical role in proper axon guidance in both central and peripheral axon tracts. Implicated in neuronal migration.
P20029	Endoplasmic reticulum chaperone BiP	72,377	61	3	5.07	7	R.IINEPTAAAIAYGLDK.R K.SQIFSTASDNQPTVTIK.V +2 Deamidated (NQ) K.DNHLLGTFDLTGIPPAPR.G +Phospho (ST)	Endoplasmic reticulum; Cytoplasm	Involved in the correct folding of proteins and degradation of misfolded proteins via its interaction with DNAJC10/ERdj5, probably to facilitate the release of DNAJC10/ERdj5 from its substrate.
Q9QXV0	ProSAAS	27,254	48	1	5.68	6	R.AVPRGEAAGAVQELAR.A	Extracellular region or secreted; Golgi apparatus	Proposed be a specific endogenous inhibitor of PCSK1. ProSAAS and Big PEN-LEN, both containing the C-terminal inhibitory domain, but not the processed peptides reduce PCSK1 activity in the endoplasmic reticulum and Golgi.
**GO mice**									
P07724	Serum albumin	68,648	49	2	5.75	3	K.CSSMQK.F +Carboxymethyl (C); Oxidation (M); Phospho (ST) R.YTQKAPQVSTPTLVEAAR.N	Extracellular region or secreted	The main function of serum albumin is the regulation of the colloidal osmotic pressure of blood.
O55143	Sarcoplasmic/endoplasmic reticulum calcium ATPase 2	114,784	38	2	5.23	3	K.TVEEVLGHFGVNESTGLSLEQVKK.L K.TGTLTTNQMSVCR.M +Deamidated (NQ); Oxidation (M)	Endoplasmic reticulum	Acts as a regulator of TNFSF11-mediated Ca^2^ signaling pathways via its interaction with TMEM64 which is critical for the TNFSF11-induced CREB1 activation and mitochondrial ROS generation necessary for proper osteoclast generation.
Q3U319	E3 ubiquitin-protein ligase BRE1B	113,897	37	4	6.13	4	R.RLQDLATQLQEK.H +Deamidated (NQ) R.TNERLKVALR.S R.EVQAEIGK.L K.ARLTCPCCNTRK.K +Carboxymethyl (C); Phospho (ST)	Nucleus	H2BK120ub1 gives a specific tag for epigenetic transcriptional activation and is also prerequisite for histone H3 ’Lys-4’ and ’Lys-79’ methylation.
Q99PT1	Rho GDP-dissociation inhibitor 1	23,393	32	1	5.12	8	K.SIQEIQELDKDDESLRK.Y	Cytoplasm	Regulates the GDP/GTP exchange reaction of the Rho proteins by inhibiting the dissociation of GDP from them, and the subsequent binding of GTP to them. Retains Rho proteins such as CDC42, RAC1 and RHOA in an inactive cytosolic pool, regulating their stability and protecting them from degradation.
P70168	Importin subunit beta-1	97,122	33	1	4.68	1	R.AAVENLPTFLVELSR.V	Nucleus; Cytoplasm	Functions in nuclear protein import, either in association with an adapter protein, like an importin-alpha subunit, which binds to nuclear localization signals (NLS) in cargo substrates, or by acting as autonomous nuclear transport receptor.
P60761	Neurogranin	7492	42	2	6.54	41	MDCCTESACSKPDDDILDIPLDDPGANAAAAK.I +2 Carboxymethyl (C); Oxidation (M); Phospho (ST) MDCCTESACSKPDDDILDIPLDDPGANAAAAK.I +3 Carboxymethyl (C); Oxidation (M); Phospho (ST)	Cytoplasm; Synapse; Dendritic spine	Regulates the affinity of calmodulin for calcium. Involved in synaptic plasticity and spatial learning.
P32848	Parvalbumin alpha	11,923	59	1	5.02	23	K.TLLAAGDKDGDGKIGVEEFSTLVAES.	Cytoskeleton Cytosol Nucleus	In muscle, parvalbumin is thought to be involved in relaxation after contraction. It binds two calcium ions.
Q8R0A5	Transcription elongation factor A protein-like 3	22,455	45	2	5.33	6	R.AAEKRPAEDYVPR.K K.RPAEDYVPR.K	Nucleus	May be involved in transcriptional regulation.

Abbreviations: young wild-type mice (WY); old wild-type mice (WO); young GNMT^−/−^ mice (GY); old GNMT^−/−^ mice (GO).

**Table 2:** Top significantly enriched canonical pathways in old mice by IPA.

Ingenuity Canonical Pathways	-log(*p*-Value)	Ratio	Molecules
GnRH Signaling	5.92	0.0729	PAK1, DNM1, GNAI2, CAMK2A, GNAS, DNM3, DNM2, CAMK2B
Clathrin-mediated Endocytosis Signaling	5.6	0.0654	DNM1, SYNJ1, ACTB, PPP3R1, DNM3, PPP3CA, DNM2
Huntington’s Disease Signaling	4.73	0.0483	DNM1, NSF, PACSIN1, HSPA1A/HSPA1B, DNM3, SNCA, DNM2
Remodeling of Epithelial Adherens Junctions	4.57	0.133	DNM1, ACTB, DNM3, DNM2
Role of NFAT in Cardiac Hypertrophy	4.02	0.0469	GNAI2, CAMK2A, GNAS, PPP3R1, PPP3CA, CAMK2B
GM-CSF Signaling	3.68	0.08	CAMK2A, PPP3R1, PPP3CA, CAMK2B
cAMP-mediated signaling	3.46	0.037	GNAI2, CAMK2A, GNAS, PPP3R1, PPP3CA, CAMK2B
B Cell Receptor Signaling	3.4	0.0467	CAMK2A, SYNJ1, PPP3R1, PPP3CA, CAMK2B
Breast Cancer Regulation by Stathmin1	3.36	0.0459	STMN1, GNAI2, CAMK2A, GNAS, CAMK2B
iCOS-iCOSL Signaling in T Helper Cells	3.34	0.0656	CAMK2A, PPP3R1, PPP3CA, CAMK2B
nNOS Signaling in Neurons	3.22	0.107	CAMK2A, PPP3R1, PPP3CA
PKCθ Signaling in T Lymphocytes	3.21	0.0606	CAMK2A, PPP3R1, PPP3CA, CAMK2B
Gap Junction Signaling	3.18	0.042	GNAI2, GNAS, ACTB, PPP3R1, PPP3CA
Protein Kinase A Signaling	3.17	0.0328	GNAI2, CAMK2A, GNAS, PPP3R1, PPP3CA, CAMK2B
fMLP Signaling in Neutrophils	3.09	0.0563	GNAI2, GNAS, PPP3R1, PPP3CA
Crosstalk between Dendritic Cells and Natural Killer Cells	3.01	0.0909	CAMK2A, ACTB, CAMK2B
PI3K Signaling in B Lymphocytes	2.98	0.0526	CAMK2A, PPP3R1, PPP3CA, CAMK2B
Synaptic Long Term Potentiation	2.96	0.0519	CAMK2A, PPP3R1, PPP3CA, CAMK2B
Chemokine Signaling	2.83	0.0789	GNAI2, CAMK2A, CAMK2B
Role of NFAT in Regulation of the Immune Response	2.8	0.0471	GNAI2, GNAS, PPP3R1, PPP3CA
Melatonin Signaling	2.73	0.0732	GNAI2, CAMK2A, CAMK2B
Dopamine-DARPP32 Feedback in cAMP Signaling	2.57	0.0408	GNAI2, GNAS, PPP3R1, PPP3CA
GABA Receptor Signaling	2.56	0.0638	DNM1, NSF, GNAS

## Data Availability

The data presented in this study are available on request from the corresponding author.

## Supplementary Materials

The following are available online at https://www.mdpi.com/1422-0067/22/6/3278/s1. Figure S1: H&E stain and representative IHC images of the cerebrum samples for ADNP, SMP30, Caspase 3 and RIPK1. Figure S2: Brain lysates from WY, WO, GY and GO mice were collected, and levels of RIPK1 were analyzed by Western blotting.

## Abbreviations

Wt	wild-type mice
GNMT	glycine N-methyltransferase
WY	young wild-type mice
WO	older wild-type mice
GY	young GNMT^−/−^ mice
GO	old GNMT^−/−^ mice

## References

[1] Santos A.L., Lindner A.B. (2017). Protein Posttranslational Modifications: Roles in Aging and Age-Related Disease. Oxid. Med. Cell. Longev..

[2] López-Otín C., Blasco M.A., Partridge L., Serrano M., Kroemer G. (2013). The hallmarks of aging. Cell.

[3] Walters R.O., Arias E., Diaz A., Burgos E.S., Guan F., Tiano S., Mao K., Green C.L., Qiu Y., Shah H. (2018). Sarcosine Is Uniquely Modulated by Aging and Dietary Restriction in Rodents and Humans. Cell Rep..

[4] Obata F., Miura M. (2015). Enhancing S-adenosyl-methionine catabolism extends Drosophila lifespan. Nat. Commun..

[5] Kerr S.J. (1972). Competing methyltransferase systems. J. Biol. Chem..

[6] Luka Z., Mudd S.H., Wagner C. (2009). Glycine N-methyltransferase and regulation of S-adenosylmethionine levels. J. Biol. Chem..

[7] Yeo E.J., Wagner C. (1992). Purification and properties of pancreatic glycine N-methyltransferase. J. Biol. Chem..

[8] DebRoy S., Kramarenko I.I., Ghose S., Oleinik N.V., Krupenko S.A., Krupenko N.I. (2013). A novel tumor suppressor function of glycine N-methyltransferase is independent of its catalytic activity but requires nuclear localization. PLoS ONE.

[9] Horgusluoglu E., Nudelman K., Nho K., Saykin A.J. (2017). Adult neurogenesis and neurodegenerative diseases: A systems biology perspective. Am. J. Med. Genet. B Neuropsychiatr. Genet..

[10] Seibert V., Ebert M.P., Buschmann T. (2005). Advances in clinical cancer proteomics: SELDI-ToF-mass spectrometry and biomarker discovery. Brief Funct. Genomic. Proteomic.

[11] Yang M.H., Chen M., Mo H.H., Tsai W.C., Chang Y.C., Chang C.C., Chen K.C., Wu H.Y., Yuan C.H., Lee C.H. (2020). Utilizing Experimental Mouse Model to Identify Effectors of Hepatocellular Carcinoma Induced by HBx Antigen. Cancers.

[12] Horvatovich P., Végvári Á., Saul J., Park J.G., Qiu J., Syring M., Pirrotte P., Petritis K., Tegeler T.J., Aziz M. (2015). In Vitro Transcription/Translation System: A Versatile Tool in the Search for Missing Proteins. J. Proteome Res..

[13] Wang Y.C., Lin W.L., Lin Y.J., Tang F.Y., Chen Y.M., Chiang E.P. (2014). A novel role of the tumor suppressor GNMT in cellular defense against DNA damage. Int. J. Cancer.

[14] Yang C.P., Wang H.A., Tsai T.H., Fan A., Hsu C.L., Chen C.J., Hong C.J., Chen Y.M. (2012). Characterization of the neuropsychological phenotype of glycine N-methyltransferase-/- mice and evaluation of its responses to clozapine and sarcosine treatments. Eur. Neuropsychopharmacol..

[15] Popa L.S., Streng M.L., Ebner T.J. (2019). Purkinje Cell Representations of Behavior: Diary of a Busy Neuron. Neuroscientist.

[16] Sassoè-Pognetto M., Patrizi A. (2017). The Purkinje cell as a model of synaptogenesis and synaptic specificity. Brain Res. Bull..

[17] Son T.G., Park H.R., Kim S.J., Kim K., Kim M.S., Ishigami A., Handa S., Maruyama N., Chung H.Y., Lee J. (2009). Senescence marker protein 30 is up-regulated in kainate-induced hippocampal damage through ERK-mediated astrocytosis. J. Neurosci. Res..

[18] Fujita T., Shirasawa T., Uchida K., Maruyama N. (1996). Gene regulation of senescence marker protein-30 (SMP30): Coordinated up-regulation with tissue maturation and gradual down-regulation with aging. Mech. Ageing Dev..

[19] Son T.G., Zou Y., Jung K.J., Yu B.P., Ishigami A., Maruyama N., Lee J. (2006). SMP30 deficiency causes increased oxidative stress in brain. Mech. Ageing Dev..

[20] Gennet N., Herden C., Bubb V.J., Quinn J.P., Kipar A. (2008). Expression of activity-dependent neuroprotective protein in the brain of adult rats. Histol. Histopathol..

[21] Cappuyns E., Huyghebaert J., Vandeweyer G., Kooy R.F. (2018). Mutations in ADNP affect expression and subcellular localization of the protein. Cell Cycle.

[22] Mollinedo P., Kapitansky O., Gonzalez-Lamuño D., Zaslavsky A., Real P., Gozes I., Gandarillas A., Fernandez-Luna J.L. (2019). Cellular and animal models of skin alterations in the autism-related ADNP syndrome. Sci. Rep..

[23] Furman S., Steingart R.A., Mandel S., Hauser J.M., Brenneman D.E., Gozes I. (2004). Subcellular localization and secretion of activity-dependent neuroprotective protein in astrocytes. Neuron Glia Biol..

[24] Pinhasov A., Mandel S., Torchinsky A., Giladi E., Pittel Z., Goldsweig A.M., Servoss S.J., Brenneman D.E., Gozes I. (2003). Activity-dependent neuroprotective protein: A novel gene essential for brain formation. Brain Res. Dev. Brain Res..

[25] Vulih-Shultzman I., Pinhasov A., Mandel S., Grigoriadis N., Touloumi O., Pittel Z., Gozes I. (2007). Activity-dependent neuroprotective protein snippet NAP reduces tau hyperphosphorylation and enhances learning in a novel transgenic mouse model. J. Pharmacol. Exp. Ther..

[26] Yang M.H., Chen S.C., Lin Y.F., Lee Y.C., Huang M.Y., Chen K.C., Wu H.Y., Lin P.C., Gozes I., Tyan Y.C. (2019). Reduction of aluminum ion neurotoxicity through a small peptide application—NAP treatment of Alzheimer’s disease. J. Food Drug Anal..

[27] Mifflin L., Ofengeim D., Yuan J. (2020). Receptor-interacting protein kinase 1 (RIPK1) as a therapeutic target. Nat. Rev. Drug Discov..

[28] Duan S., Wang X., Chen G., Quan C., Qu S., Tong J. (2018). Inhibiting RIPK1 Limits Neuroinflammation and Alleviates Postoperative Cognitive Impairments in D-Galactose-Induced Aged Mice. Front. Behav. Neurosci..

[29] Ofengeim D., Mazzitelli S., Ito Y., DeWitt J.P., Mifflin L., Zou C., Das S., Adiconis X., Chen H., Zhu H. (2017). RIPK1 mediates a disease-associated microglial response in Alzheimer’s disease. Proc. Natl. Acad. Sci. USA.

[30] Chen M., Yang M.H., Chang M.M., Tyan Y.C., Chen Y.A. (2019). Tumor suppressor gene glycine N-methyltransferase and its potential in liver disorders and hepatocellular carcinoma. Toxicol. Appl. Pharmacol..

[31] Yuan J., Amin P., Ofengeim D. (2019). Necroptosis and RIPK1-mediated neuroinflammation in CNS diseases. Nat. Rev. Neurosci..

[32] Snigdha S., Smith E.D., Prieto G.A., Cotman C.W. (2012). Caspase-3 activation as a bifurcation point between plasticity and cell death. Neurosci. Bull..

[33] Louneva N., Cohen J.W., Han L.Y., Talbot K., Wilson R.S., Bennett D.A., Trojanowski J.Q., Arnold S.E. (2008). Caspase-3 is enriched in postsynaptic densities and increased in Alzheimer’s disease. Am. J. Pathol..

[34] Shimohama S., Tanino H., Fujimoto S. (1999). Changes in caspase expression in Alzheimer’s disease: Comparison with development and aging. Biochem. Biophys. Res. Commun..

[35] Ghoumari A.M., Wehrlé R., Bernard O., Sotelo C., Dusart I. (2000). Implication of Bcl-2 and Caspase-3 in age-related Purkinje cell death in murine organotypic culture: An in vitro model to study apoptosis. Eur. J. Neurosci..

[36] Kim K., Lee S.G., Kegelman T.P., Su Z.Z., Das S.K., Dash R., Dasgupta S., Barral P.M., Hedvat M., Diaz P. (2011). Role of excitatory amino acid transporter-2 (EAAT2) and glutamate in neurodegeneration: Opportunities for developing novel therapeutics. J. Cell. Physiol..

[37] Woltjer R.L., Duerson K., Fullmer J.M., Mookherjee P., Ryan A.M., Montine T.J., Kaye J.A., Quinn J.F., Silbert L., Erten-Lyons D. (2010). Aberrant detergent-insoluble excitatory amino acid transporter 2 accumulates in Alzheimer disease. J. Neuropathol. Exp. Neurol..

[38] Thai D.R. (2002). Excitatory amino acid transporter EAAT-2 in tangle-bearing neurons in Alzheimer’s disease. Brain Pathol..

[39] Bodhinathan K., Kumar A., Foster T.C. (2010). Intracellular redox state alters NMDA receptor response during aging through Ca^2^/calmodulin-dependent protein kinase II. J. Neurosci..

[40] Zhang G.R., Cheng X.R., Zhou W.X., Zhang Y.X. (2009). Age-related expression of calcium/calmodulin-dependent protein kinase II A in the hippocampus and cerebral cortex of senescence accelerated mouse prone/8 mice is modulated by anti-Alzheimer’s disease drugs. Neuroscience.

[41] Warner H.R. (1994). Superoxide dismutase, aging, and degenerative disease. Free Radic. Biol. Med..

[42] De Haan J.B., Newman J.D., Kola I. (1992). Cu/Zn superoxide dismutase mRNA and enzyme activity, and susceptibility to lipid peroxidation, increases with aging in murine brains. Brain Res. Mol. Brain Res..

[43] Shen J., Wang C., Ying J., Xu T., McAlinden A., O’Keefe R.J. (2019). Inhibition of 4-aminobutyrate aminotransferase protects against injury-induced osteoarthritis in mice. JCI Insight.

[44] Wang Z., Lyons B., Truscott R.J., Schey K.L. (2014). Human protein aging: Modification and crosslinking through dehydroalanine and dehydrobutyrine intermediates. Aging Cell.

[45] Sanz R.L., Ferraro G.B., Girouard M.P., Fournier A.E. (2017). Ectodomain shedding of Limbic System-Associated Membrane Protein (LSAMP) by ADAM Metallopeptidases promotes neurite outgrowth in DRG neurons. Sci. Rep..

[46] Ding X., Yu J., Yu T., Fu Y., Han J. (2006). Acupuncture regulates the aging-related changes in gene profile expression of the hippocampus in senescence-accelerated mouse (SAMP10). Neurosci. Lett..

[47] Fricker L.D., McKinzie A.A., Sun J., Curran E., Qian Y., Yan L., Patterson S.D., Courchesne P.L., Richards B., Levin N. (2000). Identification and characterization of proSAAS, a granin-like neuroendocrine peptide precursor that inhibits prohormone processing. J. Neurosci..

[48] Jarvela T.S., Lam H.A., Helwig M., Lorenzen N., Otzen D.E., McLean P.J., Maidment N.T., Lindberg I. (2016). The neural chaperone proSAAS blocks α-synuclein fibrillation and neurotoxicity. Proc. Natl. Acad. Sci. USA.

[49] Wada M., Ren C.H., Koyama S., Arawaka S., Kawakatsu S., Kimura H., Nagasawa H., Kawanami T., Kurita K., Daimon M. (2004). A human granin-like neuroendocrine peptide precursor (proSAAS) immunoreactivity in tau inclusions of Alzheimer’s disease and parkinsonism-dementia complex on Guam. Neurosci. Lett..

[50] Greenwood M.P., Greenwood M., Romanova E.V., Mecawi A.S., Paterson A., Sarenac O., Japundžić-Žigon N., Antunes-Rodrigues J., Paton J.F.R., Sweedler J.V. (2018). The effects of aging on biosynthetic processes in the rat hypothalamic osmoregulatory neuroendocrine system. Neurobiol. Aging.

[51] Skillbäck T., Delsing L., Synnergren J., Mattsson N., Janelidze S., Nägga K., Kilander L., Hicks R., Wimo A., Winblad B. (2017). CSF/serum albumin ratio in dementias: A cross-sectional study on 1861 patients. Neurobiol. Aging.

[52] Luo L. (2000). Rho GTPases in neuronal morphogenesis. Nat. Rev. Neurosci..

[53] Boettner B., Van Aelst L. (2002). The role of Rho GTPases in disease development. Gene.

[54] Yu J., Gu X., Yi S. (2016). Ingenuity Pathway Analysis of Gene Expression Profiles in Distal Nerve Stump following Nerve Injury: Insights into Wallerian Degeneration. Front. Cell Neurosci..

[55] Krämer A., Green J., Pollard J., Tugendreich S. (2014). Causal analysis approaches in Ingenuity Pathway Analysis. Bioinformatics.

[56] Wang L., Chadwick W., Park S.S., Zhou Y., Silver N., Martin B., Maudsley S. (2010). Gonadotropin-releasing hormone receptor system: Modulatory role in aging and neurodegeneration. CNS Neurol. Disord. Drug Targets.

[57] Flanagan C.A., Manilall A. (2017). Gonadotropin-Releasing Hormone (GnRH) Receptor Structure and GnRH Binding. Front. Endocrinol..

[58] Ohlsson B. (2017). Gonadotropin-Releasing Hormone and Its Role in the Enteric Nervous System. Front. Endocrinol..

[59] Balasubramanian R., Dwyer A., Seminara S.B., Pitteloud N., Kaiser U.B., Crowley W.F. (2010). Human GnRH deficiency: A unique disease model to unravel the ontogeny of GnRH neurons. Neuroendocrinology.

[60] Shaw N.D., Srouji S.S., Histed S.N., McCurnin K.E., Hall J.E. (2009). Aging attenuates the pituitary response to gonadotropin-releasing hormone. J. Clin. Endocrinol. Metab..

[61] Pastore D., Pacifici F., Dave K.R., Palmirotta R., Bellia A., Pasquantonio G., Guadagni F., Donadel G., Di Daniele N., Abete P. (2019). Age-Dependent Levels of Protein Kinase Cs in Brain: Reduction of Endogenous Mechanisms of Neuroprotection. Int. J. Mol. Sci..

[62] Kim H., Oh J.Y., Choi S.L., Nam Y.J., Jo A., Kwon A., Shin E.Y., Kim E.G., Kim H.K. (2016). Down-regulation of p21-activated serine/threonine kinase 1 is involved in loss of mesencephalic dopamine neurons. Mol. Brain.

[63] Yoo D.Y., Jung H.Y., Kim J.W., Yim H.S., Kim D.W., Nam H., Suh J.G., Choi J.H., Won M.H., Yoon Y.S. (2016). Reduction of dynamin 1 in the hippocampus of aged mice is associated with the decline in hippocampal-dependent memory. Mol. Med. Rep..

[64] Jiang S., Shao C., Tang F., Wang W., Zhu X. (2019). Dynamin-like protein 1 cleavage by calpain in Alzheimer’s disease. Aging Cell.

[65] Young L.T., Warsh J.J., Li P.P., Siu K.P., Becker L., Gilbert J., Hornykiewicz O., Kish S.J. (1991). Maturational and aging effects on guanine nucleotide binding protein immunoreactivity in human brain. Brain Res. Dev. Brain Res..

[66] De Oliveira P.G., Ramos M.L.S., Amaro A.J., Dias R.A., Vieira S.I. (2019). G_i/o_-Protein Coupled Receptors in the Aging Brain. Front. Aging Neurosci..

[67] Rose A.J., Kiens B., Richter E.A. (2006). Ca^2^-calmodulin-dependent protein kinase expression and signalling in skeletal muscle during exercise. J. Physiol..

[68] Ghosh A., Giese K.P. (2015). Calcium/calmodulin-dependent kinase II and Alzheimer’s disease. Mol. Brain.

[69] Oka M., Fujisaki N., Maruko-Otake A., Ohtake Y., Shimizu S., Saito T., Hisanaga S.I., Iijima K.M., Ando K. (2017). Ca^2^/calmodulin-dependent protein kinase II promotes neurodegeneration caused by tau phosphorylated at Ser262/356 in a transgenic Drosophila model of tauopathy. J. Biochem..

[70] Liu S.P., Li Y.S., Chen Y.J., Chiang E.P., Li A.F., Lee Y.H., Tsai T.F., Hsiao M., Huang S.F., Chen Y.M. (2007). Glycine N-methyltransferase^−/−^ mice develop chronic hepatitis and glycogen storage disease in the liver. Hepatology.

[71] Carrasco M., Rabaneda L.G., Murillo-Carretero M., Ortega-Martínez S., Martínez-Chantar M.L., Woodhoo A., Luka Z., Wagner C., Lu S.C., Mato J.M. (2014). Glycine N-methyltransferase expression in the hippocampus and its role in neurogenesis and cognitive performance. Hippocampus.

[72] Quadri P., Fragiacomo C., Pezzati R., Zanda E., Forloni G., Tettamanti M., Lucca U. (2004). Homocysteine, folate, and vitamin B-12 in mild cognitive impairment, Alzheimer disease, and vascular dementia. Am. J. Clin. Nutr..

