# Supplementary material for: Utilizing an Animal Model to Identify Brain Neurodegeneration-Related Biomarkers in Aging
Source: Int J Mol Sci. 2021 Mar 23;22(6):3278. doi: 10.3390/ijms22063278 (PMC8004625; doi:10.3390/ijms22063278)
Supplement: Supplementary file 1 [file ijms-22-03278-s001.pdf]

Supplementary Materials

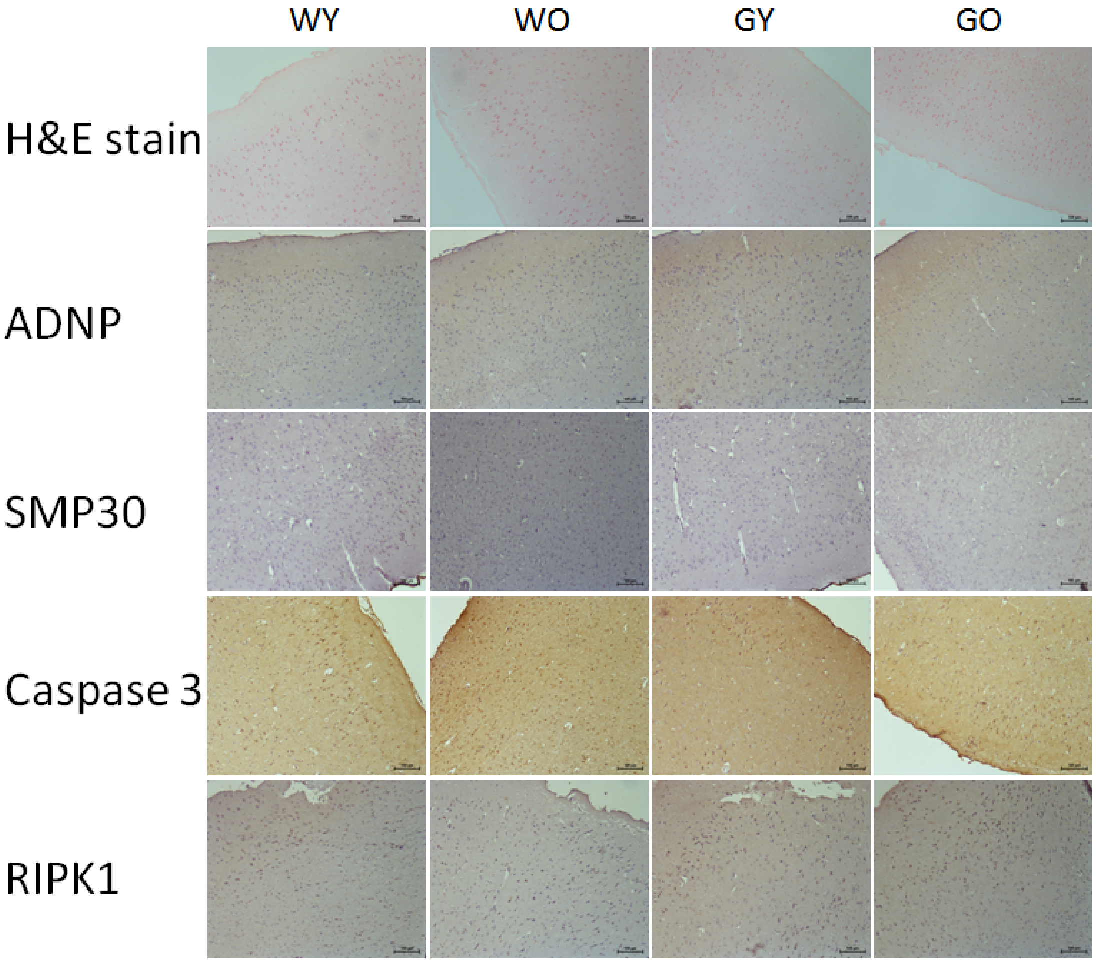

Figure S1. H&E stain and representative IHC images of the cerebrum samples for ADNP, SMP30, Caspase 3 and RIPK1. Basophilic neurons were found in the cerebral cortex. No evident difference was observed among these groups. Abbreviations: young wild-type mice (WY); old wild-type mice (WO); young GNMT-/- mice (GY); old GNMT-/- mice (GO). Scale bars: 100 μm.

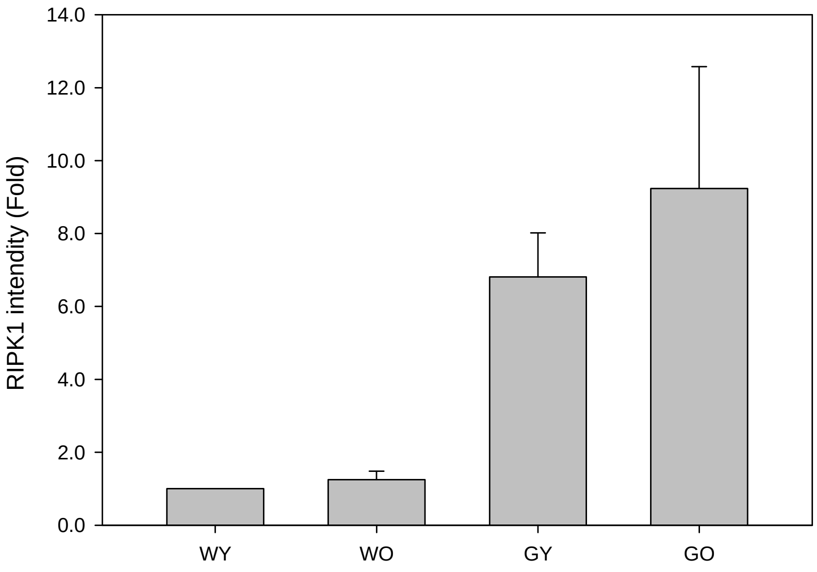

Figure S2**.** Brain lysates from WY, WO, GY and GO mice were collected, and levels of RIPK1 were analyzed by Western blotting. Beta-actin was used to normalize Western blot data (N=4). Abbreviations: young wild-type mice (WY); old wild-type mice (WO); young GNMT$^{-/-}$ mice (GY); old GNMT$^{-/-}$ mice (GO).
